# The exploration of quantum dot-molecular beacon based MoS_2_ fluorescence probing for myeloma-related Mirnas detection

**DOI:** 10.1016/j.bioactmat.2021.12.036

**Published:** 2022-01-07

**Authors:** Jing Jing Wang, Ying Liu, Zhou Ding, Le Zhang, Caiqin Han, Changchun Yan, Eric Amador, Liqin Yuan, Ying Wu, Chunyuan Song, Ying Liu, Wei Chen

**Affiliations:** a Jiangsu Key Laboratory of Advanced Laser Materials and Devices, School of Physics and Electronic Engineering, Jiangsu Normal University, Xuzhou, 221116, China; b Jiangsu Key Laboratory for Biosensors, Institute of Advanced Materials (IAM), Nanjing University of Posts & Telecommunications, Nanjing, 210023, China; c Department of Physics, The University of Texas at Arlington, Arlington, TX, 76019-0059, USA; d Department of General Surgery, The Second Xiangya Hospital, Central South University, ChangSha, Hu'nan, 410011, China; e Medical Technology Research Centre, Chelmsford Campus, Anglia Ruskin University, Chelmsford, CM1 1SQ, UK

**Keywords:** Multiple myeloma, miRNA-155, miRNA-150, Duplex-specific nuclease (DSN), Quantum dot, MoS_2_, Molecular beacon

## Abstract

Highly sensitive and reliable detection of multiple myeloma remains a major challenge in liquid biopsy. Herein, for the first time, quantum dot-molecular beacon (QD-MB) functionalized MoS_2_ (QD-MB @MoS_2_) fluorescent probes were designed for the dual detection of multiple myeloma (MM)-related miRNA-155 and miRNA-150. The results indicate that the two probes can effectively detect miRNA-155 and miRNA-150 simultaneously with satisfactory recovery rates, and the limit of detections (LODs) of miRNA-155 and miRNA-150 in human serum are low to 7.19 fM and 5.84 fM, respectively. These results indicate that our method is the most sensitive detection so far reported and that the designed fluorescent probes with signal amplification strategies can achieve highly sensitive detection of MM-related miRNAs for MM diagnosis.

## Highlights

• Novel quantum dot-molecular beacon functionalized MoS_2_ (QD-MB@MoS_2_) fluorescent probes were designed and fabricated.
• The dual detection of miRNA-155 and miRNA-150 with high sensitivity, low detection limit and high recovery was realized.
• The fluorescence probes have a great influence on the fluorescence quenching efficiency and the sensitivity.
• The new MB@MoS_2_ fluorescent probe and dual detection strategy provide a valuable tool for the detection of miRNA.

## Graphical abstract

**Figure:**
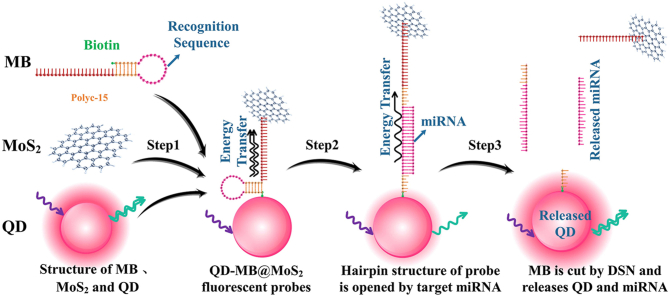

## 1 Introduction

Fluorescent probes are tracers that connect to the target substances, which are wildly used in the field of biosensing due to their sensitivity and convenience for practical applications [1]. The fluorescence probes generally include three parts: the recognition group, the connecting part, and fluorescence emitters. The function of the recognition group is to identify the target substances. The connecting part connects the recognition group and the fluorescent group, and the fluorescent groups are a component that can emit fluorescence under light excitations. So far, a variety of DNA structures have been utilized to develop DNA-based fluorescent probes, such as single-stranded DNA probes [2], double-stranded DNA probes [3], molecular beacon (MB) [4], and other structures. Among them, MB is a kind of fluorescent probe with a hairpin structure. Comparing with the single-stranded DNA fluorescent probe, MB has the advantages of high sensitivity and strong specificity. MB's hairpin structure can retain good structural stability during the detection process and can be easily designed as a probe structure to restore fluorescence by opening the hairpin structure. The regular MBs are hairpin-structured single-stranded nucleic acids that are usually labeled with fluorescence organic dyes. MBs labeled with organic dyes generally have a broad emission band with a poor photostability and easy photobleaching, which are not desirable for the development of stable and multivariate detection probes. Quantum dot (QD) is a new type of fluorescence materials with distinct advantages of strong intensity, high stability, strong anti-bleaching ability, wide excitation spectrum, narrow emission spectrum, and adjustable emission wavelength [[5], [6], [7], [8], [9]]. The effective bandgap of QDs is increased with the decrease of particle radius, resulting in a blue shift of their absorption and emission [10]. Therefore, the position of their excitation and emission can be adjusted only by changing their size. Moreover, the advantage of a wide excitation spectrum can realize the simultaneous excitation of different QDs under the same excitation. Because the fluorescence spectrum of QDs is relatively narrow, the fluorescence peaks of multiple QDs excited at the same time are not easy to overlap, which can prevent the crosstalk and interruption. Overall, these excellent fluorescence characteristics of QDs make it possible to realize simultaneous detection of biomolecules.

MicroRNA (miRNA) is a type of single-stranded RNA of 21–25 bases [11,12], which is an important regulator of cell proliferation, division, differentiation, and apoptosis since it regulates the expression level of various DNA genes after transcription [13]. The abnormal expression of specific miRNAs is related to many diseases (such as cancer, tumors, and diabetes) [[14], [15], [16]]. Therefore, miRNAs are considered to the biomarkers for important diseases like cancer [[17], [18], [19]], for example, Shen [20] et al. showed that miRNA-202 in the bone marrow microenvironment can negatively regulate BAFF by inhibiting multiple myeloma (MM) cell survival, growth, and adhesion. Long [21] et al. found that miRNA-765 plays a carcinogenic role in MM progression by directly targeting Sox6. In addition, a miRNA such as miRNA-155 may be involved in the regulation of a variety of diseases, i.e. not only regulates MM, but also relate to the production of colorectal cancer, cardiovascular and other diseases [[22], [23], [24], [25]]. Previous studies have shown that a single miRNA marker cannot reflect the specificity of a particular disease because multiple miRNA markers are often presented in the same disease. Therefore, the detection of a single miRNA biomarker is not reliable and accurate for MM diagnosis. This means that the simultaneous detection of multiple MM-related miRNAs with high sensitivity and specificity is required. However, the current high-sensitive probes and strategies for multiple miRNAs detection of MM are relatively limited [[2], [3], [4],^26^]. Therefore, more and better methods need to be explored.

**Fig. 1:**
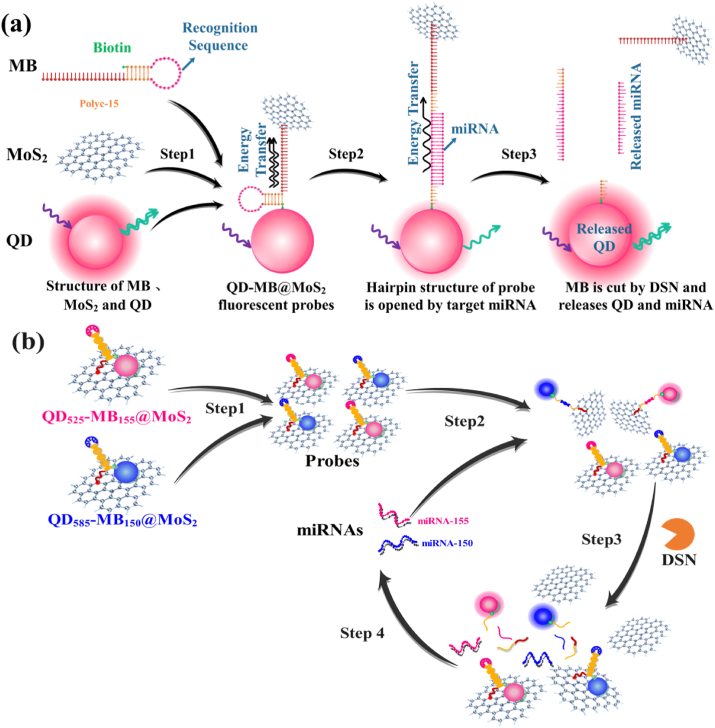
Schematic illustration of dual miRNA detection by QD-MBs functionalized MoS_2_ (QD-MB@MoS_2_) fluorescent probes. (a) Detection schematic of miRNA with QD-MB@MoS_2_ fluorescent probes and DSN-assisted signal amplification strategy. (b) Strategy of dual detection of miRNA-155 and miRNA-150 by QD-MB@MoS_2_ fluorescent probes and DSN-assisted signal amplification.

In this paper, two types of quantum dot-molecular beacons (QD-MB) were prepared on MoS_2_ monolayer nanosheet to obtain two QD-MB functionalized MoS_2_ fluorescent probes (QD_525_-MB_155_@MoS_2_ and QD_585_-MB_150_@MoS_2_). The two probes were used for the detection of MiRNA-155 and miRNA-150 simultaneously by duplex-specific nuclease (DSN)-assisted signal amplification strategy. The design principle and preparation process are shown in Fig. 1. The 3′ of MB is labeled with biotin and the 5′ was modified with 15 cytosines (PolyC15). The biotin can be coupled with the streptavidin on the surface of QDs, and PolyC15 can absorb on MoS_2_ nanosheets by van der Waals force [27] (Fig. 1(a), Step 1). At this time, QD is moving closer to MoS_2_, resulting in significant quenching of QD fluorescence. In the presence of target miRNA, the hybridization of miRNA and MB results in the formation of DNA/RNA heteroduplex, leading to the QD moving away from the MoS_2_ nanosheet and partially restoring the fluorescence of QD (Fig. 1(a), step 2). In the following step with the assistantance of the DSN molecule, the MB in the MB/RNA heteroduplex can be cut into fragments by DSN, resulting in the release of QDs and miRNAs from MoS_2_ nanosheets (Fig. 1, step 3), which enhances the fluorescence of the QD and allows the target miRNA circulated in the cycling signal amplification (Fig. 1(b), step 4). The proposed QD-MB@MoS_2_ probes and the corresponding DSN-assisted signal amplification strategy can be used for simultaneous detection of miRNA-155 and miRNA-150 in human serum with high sensitivity and selectivity, which can provide a convenient and reliable tool for clinic MM diagnosis.

## 2 Materials and methods

### 2.1 Materials and instruments

The miRNA-155, the mismatched sequences (mismatching one base (M1-155) or two bases (M2-155)), and the completely noncomplementary bases (UM-155) relative to miRNA-155, miRNA-150, the mismatched sequences with one (M1-150) or two bases (M2-150) or completely noncomplementary bases (UM-150) relative to miRNA-150, and MBs for miRNA-155 (MB155) and miRNA-150 (MB150) were purchased from Sangon Biotech Co. Ltd. (Shanghai, China). The corresponding base sequences are shown in Table 1. The streptavidin-modified QD525 (fluorescence emission at 525 nm) and QD585 (fluorescence emission at 585 nm) were purchased from Wuhan Jiayuan Quantum Technology Development Co., Ltd. (Wuhan, China). The RNAse inhibitor and monolayer MoS_2_ nanosheet were purchased from HaiGeHe Co. Ltd. (Harbin, China) and Nanjing XFNANO Material Technology Co., Ltd. (Nanjing, China), respectively. The duplex-specific nuclease (DSN) was provided by Newborn Co. Ltd. (Shenzhen, China). HEPES buffer was obtained from Procell Life Science Technology Co., Ltd. (Wuhan, China). The 0.1% diethylpyrocarbonate (DEPC) was purchased from Beijing Ligen Biotechnology Co., Ltd. (Beijing, China). Tris-Hcl, Tween-20, and BSA were purchased from Beijing Soleibao Co., Ltd. (Beijing). Human serum was purchased from Biosharp Co., Ltd. (Shenzhen, China). The ultrapure water used in this work was produced by a water purification system (H20BASIC-B, Sartorius, Germany).

**Table 1:** Nucleotide sequences of MBs and miRNAs.

Names	Sequences (5′-3′)
miRNA-155	UUAAUGCUAAUCGUGAUAGGGGU
MB_525_	CCCCCCCCCCCCCCCCCCCCATAGCGACCCCTATCACGATTAGCATTAACGCTAT-biotin
M1-155	UUAAGGCUAAUCGUGAUAGGGGU
M2-155	UUAAGGCUAAUAGUGAUAGGGGU
UM-155	AATTACGATTAGCACTATCCCCA
miRNA-150	UCUCCCAACCCUUGUACCAGUG
MB_585_	CCCCCCCCCCCCCCCCCCCCATAGCGCACTGGTACAAGGGTTGGGAGACGCTAT-biotin
M1-150	UCUCACAACCCUUGUACCAGUG
M2-150	UCUCACAACCCGUGUACCAGUG
UM-150	AGAGGGTTGGGAACATGGTCAG

A pressure cooker (GI-54DWS, Shanghai Danding International Trade Co., Ltd., China) is used to autoclave all pipette tips and reagents. Fluorescence was measured by a fluorescence spectrometer (F-4600, Hitachi, Japan). The morphology of QD was imaged by a transmission electron microscope (TEM, FEI TECNAI G2 F20).

### 2.2 Preparation of QD-MBs

22.5 μL (1 μM) quantum dot (QD_525_ or QD_585_) solution and 22.5 μL (10 μM) MB solution were injected into a centrifuge tube containing 200 μL blocking solution (including 0.2 mg/mL BSA and 0.1% Tween-20) and 600 μL Tris-Hcl buffer, followed by shaking for 2 h. The solution was then transferred to a 100 K ultrafiltration tube centrifugally (4000 rpm for 3 min) to remove the free MBs three times. The pure QD-MBs dispersed in 100 μL TrisHcl buffer and 45 μl blocking solution were obtained and stored in 4 °C for future use. The above operations were carried out at room temperature.

### 2.3 Preparation of QD-MB@MoS2 fluorescent probes

The QD-MBs were mixed with monolayer MoS_2_ nanosheets to prepare QD-MB@MoS_2_ fluorescent probes. Briefly, 22.5 μL QD-MBs, 67.5 μL blocking solution, and 200 μL 0.64 μM MoS_2_ nanosheets were added into 250 μL buffer (5 mM Tris-HCl, 1 M NaCl, pH = 7.4), followed by diluting the mixture to 500 μL with ultrapure water and shaking it for 1 h. The obtained mixture was centrifugally purified (4000 rpm for 3 min) to obtain pure QD-MB@MoS_2_ by re-dispersing the sediments in 125 μL TrisHcl buffer and 75 μL blocking solution. The QD-MB@MoS_2_ solution was stored at 4 °C. The above operations are all carried out at room temperature. The fluorescent probe QD_525_-MB_155_@MoS_2_ of miRNA-155 and the fluorescent probe QD_585_-MB_150_@MoS_2_ of miRNA-150 are produced according to the above steps, separately.

### 2.4 Dual detection of miRNA-155 and miRNA-150 in buffer

The two fluorescence probes obtained in the previous step were mixed in a volume ratio of 1:1 to prepare QD-MB@MoS_2_ probes for dual detection of miRNA-155 and miRNA-150 (Fig. 1(b), step 3). Using HEPES buffer as the diluent, miRNA-155 and miRNA-150 were diluted into different concentrations (10 fM, 100 fM, 1 pM, 10 pM, 100 pM, and 1 nM, respectively). Then, 20 μL miRNA-155 and 20 μL miRNA-150 solutions with the same concentration were mixed to prepare the miRNAs mixture for the test. Subsequently, the miRNAs mixture was added into 100 μL QD-MB@MoS_2_ probes. After incubating for 30 min, 0.3 U of DSN was added into the mixture and incubated at 55 °C for 40 min. Finally, the fluorescence of the mixture was recorded.

### 2.5 Dual detection of miRNA-155 and miRNA-150 in 10% human serum

To test for practical applications, miRNA-155 and miRNA-150 were spiked in 10% human serum with different concentrations (10 fM, 100 fM, 1 pM, 10 pM, 100 pM, and 1 nM) and tested by the QD-MB@MoS_2_ probes following the above-mentioned sensing strategy. Besides, the mixture including an equal concentration of miRNA-155 and miRNA-150 (80 fM, 200 fM, 50 pM, and 540 pM, respectively) were also tested to characterize the recovery rate.

## 3 Results and discussion

### 3.1 Characterization of quantum dots and MoS_2_

**Fig. 2:**
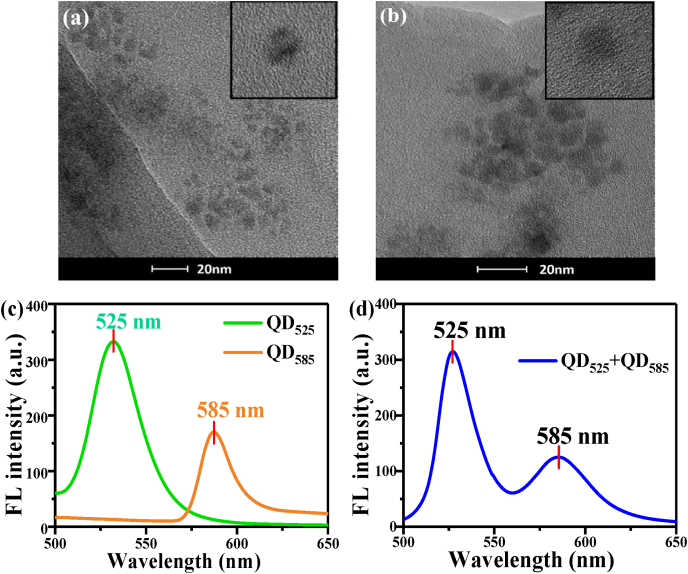
TEM images and fluorescence spectra of QD_525_ and QD_585_. (a) TEM image of QD_525_. (b) TEM image of QD_585_. (c) Fluorescence spectra of QD_525_ and QD_585_, respectively. (d) Fluorescence spectra of the mixture of QD_525_ and QD_585_.

The TEM pictures of CdSe/ZnS quantum dots QD_525_ and QD_585_ are shown in Fig. 2 (a) and (b). It can be seen that the quantum dots QD_525_ and QD_585_ maintain a relatively good spherical shape and dispersion. According to the information provided by the merchant, the diameters of QD_525_ and QD_585_ are 47.5 and 48.5 nm, respectively, and the quantum yields are 91% and 89%, respectively. The fluorescence spectra of CdSe/ZnS quantum dots QD_525_ and QD_585_ are shown in Fig. 2(c). Under the excitation of 365 nm, the fluorescence e mission peaks of QD_525_ and QD_585_ are located at 525 nm and 585 nm, respectively. Fig. 2(d) shows the fluorescence spectrum of the mixture solution containing two quantum dots with a volume ratio of 1:1. The emission peak position and fluorescence intensity of QD_525_ and QD_585_ are consistent with the ones of isolated QD_525_ and QD_585_ in Fig. 2(c), and the emission peaks of QD_525_ and QD_585_ have a shift of 60 nm, which indicates that fluorescence of the two quantum dots can be distinguished clearly.

**Fig. 3:**
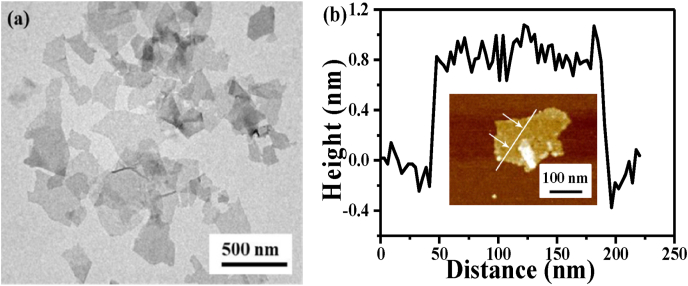
(a) TEM image of MoS_2_. (b) Height profile along the line shown in insert map of (b).

The TEM image is shown in Fig. 3(a) illustrates the monolayer morphology of MoS2 and the average size is about 237 ± 42 nm. The AFM characterizations shown in Fig. 3(b) indicate the monolayer structure of MoS2 nanosheets with height about 1.0 nm. The direct bandgap width of monolayer MoS_2_ is about 1.75 eV [[28], [29], [30]], and the corresponding cut-off adsorption wavelength can be calculated to be 708.5 nm. This means that MoS_2_ can absorb light with a wavelength less than 708.5 nm. The monolayer MoS_2_ has five absorption peaks in the Ultraviolet–Visible wave band, which are located near 270 nm, 405 nm, 448 nm, 620 nm, and 670 nm respectively [30,31]. The overlapping spectrum of the four absorption peaks in the visible wave band almost covers the whole visible wave band, resulting in its absorption of the entirety of the visible light. Therefore, the appearance of the monolayer MoS_2_ dispersant is black. The positions of the fluorescence emission peaks of QDs selected in this paper are 525 nm and 585 nm, which overlap well with the absorption spectrum of monolayer MoS_2_ and meet the conditions of energy transfer quenching [32].

### 3.2 Fluorescence characterization of QD_525_-MB_155_@MoS_2_ and QD_585_-MB_150_@MoS_2_ probes

**Fig. 4:**
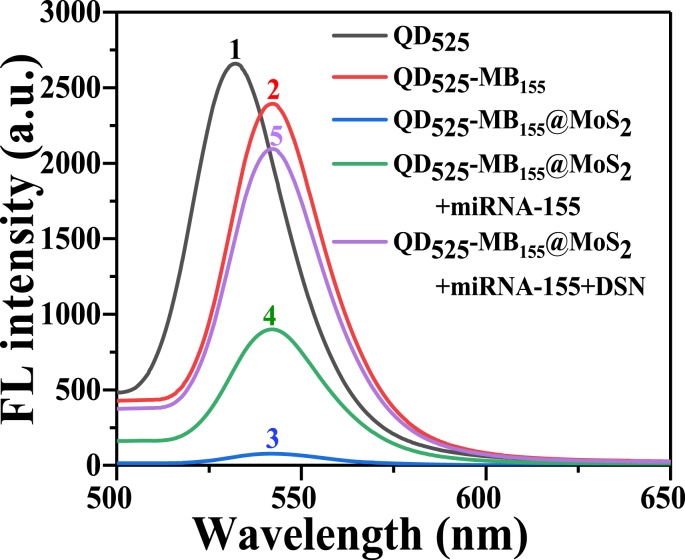
Fluorescence characterization of QD_525_ during the preparation process of QD_525_-MB_155_@MoS_2_ probes and the assay of miRNA-155.

Fig. 4(a) shows the fluorescence spectra of the QD_525_ during the preparation process of QD_525_-MB_155_@MoS_2_ probes and the assay of miRNA-155 under the excitation of 365 nm incident light. Curve No. 1 (black) is the fluorescence spectrum of free QD_525_, and the fluorescence emission peak is at 525 nm. Curve No. 2 (red) is the fluorescence spectrum of QD_525_ modified MB_155_ (QD_525_-MB_155_), and the emission peak is at 542 nm. The peak of QD_525_-MB_155_ fluorescence spectrum showed some red shift, which may be caused by the change of surface charge state and charge transfer caused by MB modification [[33], [34], [35], [36]].

Curve No. 3 (blue) is the fluorescence spectrum obtained after adding MoS_2_ to QD_525_-MB_155_, i.e. the fluorescence spectrum of QD_525_-MB_155_@MoS_2_ probes. The position of the fluorescence peak sticks at 542 nm, but the peak intensity is very weak, which indicates that the MoS_2_ has been coupled to the 5′-end of the MB, and the fluorescence of QD_525_ is quenched due to the influence of adjacent MoS_2_. At this time, the maximum distance between QD and MoS_2_ is about 8.8 nm (Calculated based on the base sequence number of MB). According to the fluorescence quenching theory, it is judged that the mechanism of MoS_2_ quenching QD fluorescence is Förster resonance energy transfer (FRET) which generally occurs between donors (fluorescent group) and acceptors (fluorescent quencher) with a spacing of 1–10 nm. When the vibration frequencies of the two dipoles are the same, the donor oscillates due to the absorption of radiation energy, and the receptor oscillates with the oscillation of the donor, that is, resonance occurs. As a result, the donor transfers the absorbed energy to the receptor and loses the ability to radiate energy, that is, the fluorescence quenching effect occurs. According to the calculation method provided by Cristobal [35], the energy transfer efficiency is calculated to be 0.9675 (The fluorescence intensity of curve 2 is taken as the original fluorescence intensity without acceptors).

Curve No. 4 (green) is the fluorescence spectrum of QD_525_-MB_155_@MoS_2_ after incubation with 1 nM miRNA-155 (QD_525_-MB_155_@MoS_2_ +miRNA-155). Compared with the fluorescence spectrum of the QD_525_-MB_155_@MoS_2_ (No. 3), the fluorescence peak intensity at 542 nm is significantly enhanced. This is due to the hybridization of miRNA-155 and MB on the QD_525_-MB_155_@MoS_2_ probe, resulting in the opening of the hairpin structure of MB_155_, which caused QD_525_ to move away from the MoS_2_ nanosheet and restore part of its fluorescence. According to the base number of MB, it is calculated that the maximum distance between QD and MoS_2_ after the MB structure is opened is about 18.7 nm, which is greater than the effective distance of FRET 10 nm. However, the fluorescence of QD has not fully recovered to the original intensity, and analysis suggests that the fluorescence quenching mechanism of energy radiation transfer has occurred [32]. As an energy donor, QD releases the excitation energy in the form of radiation and returns itself to the ground state. MoS_2_, as a receptor, absorbs the photons radiated by QD and is excited, resulting in QD fluorescence quenching. It is calculated that the energy transfer efficiency is 0.62375.

When DSN was added to cut the DNA (i.e. MB base sequence) within the DNA/RNA heteroduplex, the significantly enhanced fluorescence spectrum is shown as curve No. 5 (purple) in Fig. 4(a). The result shows that the DSN selectively and efficiently cut the MB in the double strands, which makes the QDs release from the MoS_2_ nanosheet. At the same time, miRNA-155 was also released and reused to hybridize with MBs on QD_525_-MB_155_@MoS_2_ probes, so that a DSN-assisted miRNA-155 recycling signal amplification was achieved. Theoretically, the released QD becomes a free state and can restore all fluorescence. However, MoS_2_ nanosheets still exist in the solution, and the distance between MoS_2_ and QD is far greater than 10 nm. It is believed that there is still some fluorescence quenching process of radiation energy transfer. The efficiency of energy radiation transfer is inversely proportional to the square of the distance R [32], so the recovered fluorescence intensity is much stronger than that of Curve No. 4, but it has not yet reached the recovery of all fluorescence. After being clipped by DSN, the energy transfer efficiency is reduced to 0.125. Based on the above analysis, it is believed that the mechanism of MoS_2_ quenching the fluorescence of QDs in this experiment is mainly FRET, followed by radiation energy transfer (when the distance between MoS_2_ and QD is greater than 10 nm). And this results also illustrate that the length of the designed MB base sequence has a great influence on the energy transfer efficiency, that is, it has a great influence on the fluorescence quenching effect and the detection sensitivity of fluorescent probes.

### 3.3 Optimization of preparation conditions for fluorescent probes

**Fig. 5:**
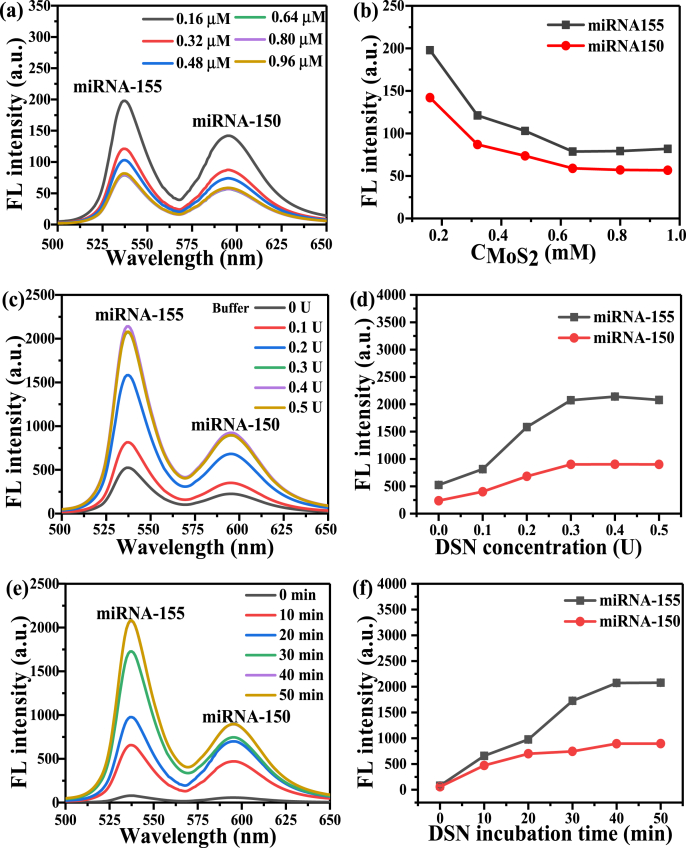
Optimization of the QD-MB@MoS_2_ and the sensing strategy. (a) Fluorescence spectra of the QD-MB@MoS_2_ prepared by different concentrations of MoS_2_ (i.e. 0.16, 0.32, 0.48, 0.64, 0.80, and 0.96 μM). (b) Plot of the relationship between the fluorescence intensity and the concentration of MoS_2_ corresponding to the spectra in (a). (c) Fluorescence spectra of the assay of 1 nM miRNA by using different concentrations of DSN (0, 0.1, 0.2, 0.3, 0.4 and 0.5 U). (d) Plot of the relationship between the fluorescence intensity and DSN concentration corresponding to the spectra in (c). (e) Fluorescence spectra of the assay of 1 nM miRNA by incubating 0.3 U DSN for different incubation times (0, 10, 20, 30, 40, and 50 min). (f) Plot of the relationship between the fluorescence intensity and the incubation time corresponding to the spectra in (e). Error bars represent standard deviations from three measurements.

To optimize the preparation of QD-MB@MoS_2_, different concentrations of MoS_2_ were used (i.e. 0.16, 0.32, 0.48, 0.64, 0.80, and 0.96 μM) to prepare QD-MB@MoS_2_ according to the protocol mentioned in the experimental section, followed by characterizing the fluorescence of the mixture. As shown in Fig. 5(a) and Fig. 5(b), the fluorescence intensities of the two fluorescent probes decreases with the increasing concentration of MoS_2_, and almost constant fluorescence quenching effect was obtained when the concentration of MoS_2_ increased to 0.64 μM, which indicates that the optimal concentration of MoS_2_ is 0.64 μM.

The dosage of DSN was optimized by fluorescence detection of 1 nM miRNA-155 and miRNA-150 by using different concentrations of DSN (i.e. 0, 0.1, 0.2, 0.3, 0.4, and 0.5 U) with the results shown in Fig. 5(c) and (d). As shown in Fig. 5(d), when the DSN concentration increases from 0 to 0.3 U, the fluorescence intensity gradually increases until it reaches a saturation value, which indicates that the optimal DSN concentration is 0.3 U. Fig. 5(e) plots the fluorescence spectra of the assay of 1 nM miRNA with 0.3 U DSN for different incubation times (0, 10, 20, 30, 40, and 50 min), and Fig. 5(f) plots the fluorescence peak intensity measured at different incubation times. It can be seen clearly that the fluorescence peak intensity increases to a saturation value when the incubation time increases from 0 min to 40 min, indicating that the optimal incubation time of DSN is 40 min.

### 3.4 Dual detection of miRNAs in buffer

**Fig. 6:**
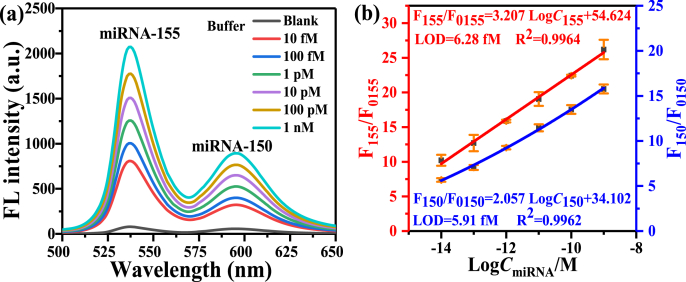
(a) Fluorescence spectra of the simultaneous assay of miRNA-155 and miRNA-150 in buffer with different concentrations. (b) Plots of relationships between the fluorescence ratio and the logarithm of miRNA concentration of miRNA-155 and miRNA-150, respectively. F_0155_ and F_0150_ represent the fluorescence intensity of QD_525_-MB_155_@MoS_2_ probes and QD_585_-MB_150_@MoS_2_, respectively. F_155_ and F_150_ represent the fluorescence intensity of the simultaneous assay of miRNA-155 and miRNA-150. Error bars represent the standard deviations from three measurements.

Fig. 6(a) shows the fluorescence dual detections of miRNA-155 and miRNA-150 in buffer with different concentrations (i.e. 10 fM, 100 fM, 1 pM, 10 pM, 100 pM and 1 nM). Fig. 6(b) plots the relationships between the logarithms of miRNA-155 and miRNA-150 concentrations (logC_miRNA_) and the fluorescence intensity ratio (F/F_0_), where F_0_ represents the fluorescence intensity of probes, and F represents the fluorescence intensity of miRNA assay. It can be seen that two linear fitting curves, i.e. F_155_/F_0155_ = 54.624 + 3.207 × logC_155_ (R^2^ = 0.9964) and F_150_/F_0150_ = 34.102 + 2.057 × logC_150_ (R^2^ = 0.9962), were obtained from 10 fM to 1 nM. The calculated limit of detections (LODs) of miRNA-155 and miRNA-150 are 6.28 fM and 5.91 fM (3σ/s), respectively, where σ is the standard deviation of F/F_0_ and s is the slope of the linear equation. According to the calibration curve, miRNA-155 and miRNA-150 can be simultaneously detected qualitatively and quantitatively by the proposed fluorescence detection strategy.

### 3.5 Specificity

**Fig. 7:**
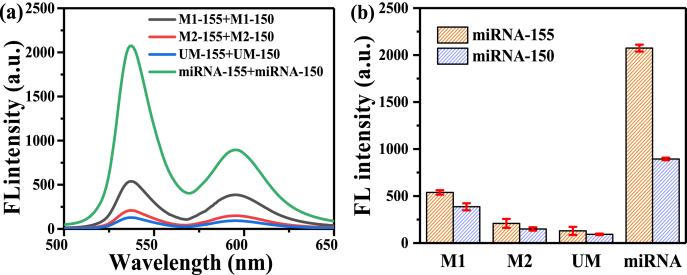
Characterization of the performance of the fluorescent probe on the simultaneous assay of miRNA-155 and miRNA-150 in the buffer. (a) Specificity characterization by recording the fluorescence spectra of the assays of 1 nM unmatched (UM), single-base mismatched miRNA-155 and miRNA-150 (M1), two bases mismatched miRNA-155 and miRNA-150 (M2), and 1 nM miRNA-155 and miRNA-150. Blank means the assay in the absence of miRNA-155 and miRNA-150. (b) Plot of the fluorescence peak intensities of the corresponding spectra shown in (a). Error bars represent standard deviations from three measurements.

To study the specifics of fluorescent probes for miRNA-155 and miRNA-150, M1-155 (single-base mismatched miRNA-155), M2-155 (two-base mismatched miRNA-155), UM-155 (unmatched miRNA-155), M1-150 (single-base mismatched miRNA-150), M2-150 (two-base mismatched miRNA-150), and UM-150 (unmatched miRNA-150) were also tested. The recorded fluorescence spectra are shown in Fig. 7, which indicates that the fluorescence signal of specific detections can be distinguished from the non-specific detections and blank. The error probabilities during RNA replication and translation are 10^−10^ and 10^−5^, respectively [[37], [38], [39]]. Therefore, the concentration of mature miRNA in the human body is much higher than that of wrong code miRNA. Even if there is a small amount of miscoded miRNA in human serum, it can still provide partial fluorescence. Therefore, we believe that the fluorescence loss caused by miscoded miRNA is less than the error value and does not affect the overall measurement level of miRNA. Therefore, the prepared fluorescent probes and sensing strategy have good specificity.

### 3.6 Dual detections of miRNAs in 10% human serum

**Fig. 8:**
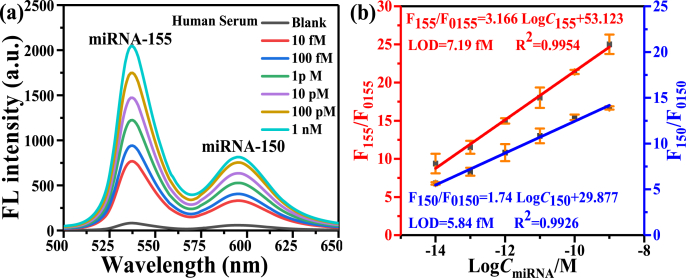
(a) Fluorescence spectra of the simultaneous assays of miRNA-155 and miRNA-150 in human serum. (b) Plot of the relationships between fluorescence ratio and logarithm of concentration of miRNA-155 and miRNA-150, respectively. Error bars represent standard deviations from three measurements.

To investigate the practical application of the proposed probes and strategy, the 10% human serums including an equal concentration of miRNA-155 and miRNA-150 at different concentrations (10 fM, 100 fM, 1 pM, 10 pM, 100 pM, and 1 nM) are prepared and fluorescence detected respectively. The fluorescence spectra of assays of miRNA-155 and miRNA-150 in human serum with different concentrations (1 nM–10 fM) is shown in Fig. 8(a). Fig. 8(b) plots the miRNA concentration-dependent fluorescence intensity relationships of miRNA-155 and miRNA-150, respectively. The relationship between F_155_/F_0155_ and logC_miRNA_ follows a linear relationship, and the calibration curve is F_155_/F_0155_ = 53.123 + 3.166 × logC_155_ (R^2^ = 0.9954). Similarly, for miRNA-150 the linear calibration curve is F_150_/F_0150_ = 29.877 + 1.74 × logC_150_ (R^2^ = 0.9954). The LODs of miRNA-155 and miRNA-150 in human serum are 7.19 fM and 5.84 fM respectively, which is very close to the LODs of miRNAs in the buffer. The results show the good anti-interference ability of probes for detecting miRNAs in complex samples. Liu Ying et al. detected the fluorescence intensity of miRNA-155 in serum of healthy people and MM patients with MB-functionalized monolayer MoS_2_ nanosheet fluorescent probe [40]. According to the experimental results, we can infer that the concentration of miRNA-155 in health people serum is about 53.7 pM, and that of miRNA-155 in MM patients serum is about 6.12 nM, which proves that the sensitivity and detection range achieved by our pattern method can meet the clinical detection requirements of miRNA.

The recovery rate was further studied by testing the mixtures including equivalent miRNA-155 and miRNA-150 spiked in 10% human serum, i.e. 80 fM, 200 fM, 50 pM, and 540 pM. According to the found value of the assay shown in Table 2 and Table 3, the recovery rate of miRNA-155 is in the range of 92.14–105.70% with the relative standard deviation (RSD) less than 7.92%, and the recovery rate of miRNA-150 is in the range of 94.03–102.6 2% with the RSD less than 8.22%, which confirms that the proposed probes and strategy can be used for detecting miRNAs in complex serum and has the clinical application prospects.

**Table 2:** Recovery of the assay of miRNA-155 in 10% human serum.

Sample	Added	Found (n = 3)	Recovery/%	RSD/%
1	80 fM	73.71 fM	92.14	5.18
2	200 fM	196.32 fM	98.16	7.92
3	50 pM	52.85 pM	105.70	4.28
4	540 pM	554.09 pM	102.61	5.30

**Table 3:** Recovery of the assay of miRNA-150 in 10% human serum.

Sample	Added	Found (n = 3)	Recovery/%	RSD/%
1	80 fM	75.22 fM	94.03	8.22
2	200 fM	198.70 fM	99.35	3.94
3	50 pM	51.31 pM	102.62	5.11
4	540 pM	551.20 pM	102.07	4.70

## 4 Conclusions

In this paper, novel quantum dot-molecular beacon functionalized MoS_2_ (QD-MB @MoS_2_) fluorescent probes were designed and fabricated for the dual detection of MM-related miRNA-155 and miRNA-150 via DSN-assisted signal amplification. The QD-MB@MoS_2_ fluorescent probes were constructed by linking polyC15-modified MBs to CdSe/ZnS quantum dots and further absorbing on monolayer MoS_2_ nanosheets. The fluorescence quenching and fluorescence recovery mechanism of the QD-MB@MoS_2_ probes used in the experiment were analyzed. The results illustrate that the mechanism of MoS_2_ quenching the fluorescence of QDs is mainly FRET, followed by radiation energy transfer (when the probes are opened or cut off), and the designed MB length has a great influence on the fluorescence quenching efficiency and the sensitivity of the fluorescent probe. Based on the fluorescent probes, a new scheme was designed and used to achieve high-sensitivity simultaneous detection of dual miRNA (miRNA-155 and miRNA-150). The results indicate that the two probes can sensitively detect miRNA-155 and miRNA-150 simultaneously with a good recovery rate, and the LODs of miRNA-155 and miRNA-150 in human serum are low to 7.19 fM and 5.84 fM respectively. These results indicate that the proposed new MB@MoS_2_ fluorescent probe and the DSN-assisted signal amplification strategy for miRNA detection not only has the characteristics of low biological toxicity, selectable wavelength and easy to realize multiplex detection, but also has the advantages of high sensitivity and low detection limit, which provide a valuable tool for clinical diagnosis of cancers and other diseases.

## CRediT authorship contribution statement

**Jing Jing Wang:** Methodology, Synthesis, measurements, Formal analysis, Investigation, Writing – original draft. **Zhou Ding:** Methodology, Synthesis, measurements, Formal analysis, Investigation, Writing – original draft. **Le Zhang:** Resources, Instruction, Funding acquisition. **Caiqin Han:** Resources, Instruction, Funding acquisition. **Eric Amador:** Instruction, Formal analysis, Validation. **Liqin Yuan:** Instruction, Formal analysis, Validation. **Ying Wu:** Resources. **Chunyuan Song:** Writing – review & editing, Validation. **Ying Liu:** Conceptualization, Supervision, Project administration, Funding acquisition, Methodology, Synthesis, measurements, Formal analysis, Investigation, Writing – original draft, Conceptualization, Supervision, Project administration, Funding acquisition, Methodology, Synthesis, measurements, Formal analysis, Investigation, Writing – original draft. **Wei Chen:** Conceptualization, Supervision, Project administration, Funding acquisition, Writing – review & editing, Validation.

## Declaration of competing interest

The authors declare that they have no known competing financial interests or personal relationships that could have appeared to influence the work reported in this article.
